# Change of Cyclosporine Absorption over the Time after Kidney Transplantation

**DOI:** 10.5812/numonthly.2437

**Published:** 2012-03-01

**Authors:** Behzad Einollahi, Mojtaba Teimoori, Zohreh Rostami

**Affiliations:** 1 Nephrology and Urology Research Center, Baqiyatallah University of Medical Sciences, Tehran, IR Iran

**Keywords:** Cyclosporine, Delayed Graft Function, Kidney Transplantation

## Abstract

**Background:**

Although the immunosuppressant cyclosporine (CsA) is widely used after kidney transplantation over the long term, there is still no firm consensus on the best way to monitor of CsA blood levels.

**Objectives:**

Cyclosporine (CsA) assay is critical for the management of renal transplant recipients due to inter– and intra–patient variation in CsA absorption and metabolism. Patients and Methods: In a retrospective cross sectional study, blood levels of CsA (through and 2 hours post dose) measured at least 5 times during 3 years post transplantation, in 7702 kidney transplant recipients from different transplant center of Tehran, IR Iran between 2008 and 2012. Cyclosporine absorption (CA) calculated C2/C0 ratio.

**Results:**

CA had a significant correlation with allograft function (P = 0.000, r =.0.285), this correlation was stronger than its relationship with C0 and C2 blood levels (P = 0.000 and P = 0.000 as well as r = 0.033 and r = 0.090, respectively). In univariate analysis during different times after transplantation, C0 and C2 blood levels significantly decreased over three years follow up (P = 0.000), (P = 0.000); While, CA reversely increases over the time (P = 0.000). In linear regression model overall CA levels had correlation with lower age of recipient (P = 0.02), hypokalemia (P = 0.001), higher level of creatinine (P = 0.02) and triglyceride (P = 0.001).

**Conclusions:**

The present study shows that CsA absorption changes trough the post-transplant time and appears to increases over time in long–term period after kidney transplantation.

## 1. Background

Although the immunosuppressant cyclosporine (CsA) is widely used after kidney transplantation over the long term, there is still no firm consensus on the best way to monitor CsA blood levels (1). It is routinely monitored by predosage blood trough level (C0) or two hours post dose level (C2). In fact, these measurements cannot certainly assess the individual biological impact of the CsA.

Optimization of CsA effect on recipient’s immune system is critical to increase short- and long-term outcomes in kidney transplants. Despite improvement in therapeutic CsA monitoring, allograft rejection and CsA nephrotoxicity are still two important problems in kidney transplant patients (2).

There is a day-to-day variability of CsA absorption (CA), described as C2/C0 (3) ratio, and inter-individual variability in drug level is highest within the absorption phase (0 to 4 h post-dose) and satisfactory CsA blood concentration in this period is important for its efficacy (4). Thus, the CsA level assays are critical for the management of renal transplant recipients due to inter- and intra-patient variations in CsA absorption and metabolism. Moreover, some researchers have suggested that a combination of C0 and C2 blood level assays may be a beneficial method for evaluating the CsA absorption profile, because it involves both the elimination and the absorption phases (C0 and C2) (5-6). The CsA absorption (C2/C0 ratio) is also practical for determining the high or low CsA absorbers (5, 6). The limited published data are available in literature in terms of CsA absorption profiling over time in renal transplant patients (5-9).

## 2. Objectives

To our knowledge, these studies are limited by relatively small sample sizes and short-term follow up. In a series of 98 kidney transplants, the absorption profile of CsA is evaluated during the first year after transplantation (5). Therefore, we conducted a retrospective study to assess the CsA absorption, as described by C2/C0 ratio, during the first three post-transplant years in a large renal transplant population.

## 3. Patients and Methods

### 3.1.Study Population

In a retrospective cross sectional study, blood levels of CsA measured in 7702 kidney transplant recipients from different transplant center of Tehran, I.R. Iran between 2008 and 2012 were analyzed. All measurements were performed in a single laboratory. Ethical approval of research was confirmed by the Local Ethics Committee of University. 

### 3.2.Immunosuppressant

Immunosuppressance was based on CsA plus mycophenolate mofetil or azathioprine and prednisolone in all patients . In most centers, CsA doses given to kidney recipients were administered mostly upon CsA trough levels. CsA measurement was assessed at different times and dose was adjusted as local protocol and in case of necessity. Our therapeutic target ranges for C0 levels were 200 to 300 ng/mL in 1 to 3 months, 100 to 250 ng/mL in 4 to 12 months and 100 to 150 ng/mL in more than 1 year after transplantation; while C2 target levels were 800 to 1000 ng/mL in months one to three after transplantation and C2 targets of 400 to 600 ng/mL for subsequent months.

### 3.3.Laboratory Data Collection

Routine laboratory investigations included creatinine (Cr), uric acid, hemoglobin (Hb), sodium, potassium, phosphorous, cholesterol (Chol), triglyceride (TG), high density lipoprotein cholesterol (HDL), low density lipoprotein cholesterol (LDL). Blood was collected in the morning after a 12-hour fasting period. The biochemical assays were performed by the same laboratory using automatic systems with various relevant methods including enzymatic method (blood urea nitrogen, Chol, TG, HDL, LDL), modification of the kinetic Jaffe reaction (Cr). All methods for biochemical assays had inter- and intraassay coefficients of variation within 5%. CsA levels were determined from whole blood using the Cobas Mira-Plus analyzer (Roche). Cyclosporine absorption (CA) was calculated by C2/C0 ratio.

### 3.4.Statistical Analysis

The SPSS version 17.0 for Windows was used in all the analysis. Quantitative variables were expressed as mean ± standard deviation (SD), while qualitative variables were shown by number and percentage. The kolmogorov-simirnov test showed that C0, C2 and calculated CA was not distributed normally; hence, Spearman’s correlation analysis was used to study correlations between CA, C0 and C2 concentrations with numeric variable such as serum Cr, age of recipient, TG, LDL, HDL and Hb. We used a formula Ln (CA) to normalize the CA distribution for Linear regression model. Linear logistic regression, backward model was used for analyze factors with the greatest explanatory effect on CA level. ANOVA model used to evaluate a change in 5 different times of C0, C2 and CA level during 3 years of follow up. We also used generalized linear model (GLM) for detecting effect of categorical variable such type of donor and gender of donor and recipient on C0, C2 and CA. A P < 0.05 was regarded as a significant level and 95% confidence interval was also considered to be a reliable estimate. We used P < 0.2 for entering variable in to linear regression model.

## 4. Results

### 4.1.Demographical Setting

A total of 7702 patients were recruited from different Transplant Centers of Iran. The mean age of recipients was 37 ± 15 years (range: 3 to 83 years); 63% male and 37% female (Table 1). The mean age of donors was 28 ± 6 years (range: 5 to 62 years); 83% male and 17% female. The majority of grafts came from living donors (84% unrelated and 8% related), whereas 8% of patients received a deceased donor graft.

**Table 1:** Relation of Baseline Characteristics of Patients With C0 and C2

	0-3, mo	4 to 6, mo	7-12, mo	2nd, y	3rd, y	P Value
Cyclosporine trough level	254.85 ± 116.16	197.60 ± 88.25	168.09 ± 78.02	153.29 ± 80.21	145.49 ± 87.25	0.000
Cyclosporine 2 hours post dose	764.90 ± 162.93	636.15 ± 127.67	575.89 ± 115.80	539.48 ± 152.75	500.55 ± 180.15	0.000
Cyclosporine absorption	3.38 ± 0.83	3.82 ± 0.90	4.05 ± 1.07	4.22 ± 1.10	4.33 ± 1.24	0.000

### 4.2.Correlation of CA and Allograft Function

CA had a significant correlation with allograft function (P = 0.000, r = 0.285), this correlation was stronger than its relationship with C0 and C2 blood levels (P = 0.000 and 0.000 as well as r = 0.033 and 0.090, respectively).

### 4.3.Univariate Analysis

In five different times after transplantation, C0 and C2 blood levels significantly decreased over three years follow up; while, CA reversely increased over the time (Table 1). We found that lower age of recipient, hypokalemia, hypernatremia, hypotriglyceridemia, anemia, higher Cr concentration and lower LDL were significantly related to CA (Table 2).

**Table 2:** Relation of Baseline Characteristics of Patients With Cyclosporine A

	Sig. (2-tailed)	Spearman’s Correlation
Age of recipient	0.000	-0.142
Hb a	0.004	-0.092
HDL a cholesterol	0.153	-0.054
LDL a cholesterol	0.039	-0.080
Triglycerides	0.000	-0.165
potassium	0.002	-0.083
phosphorus	0.000	0.099
Sodium	0.004	0.077
Creatinine	0.000	0.132
Blood urea	0.103	-0.040

^a^Abbreviations: Hb, Hemoglobin; HDl, high density lipoprotein; LDL, low density lipoprotein

### 4.4.Generalized Linear Model

**Figure 1:**
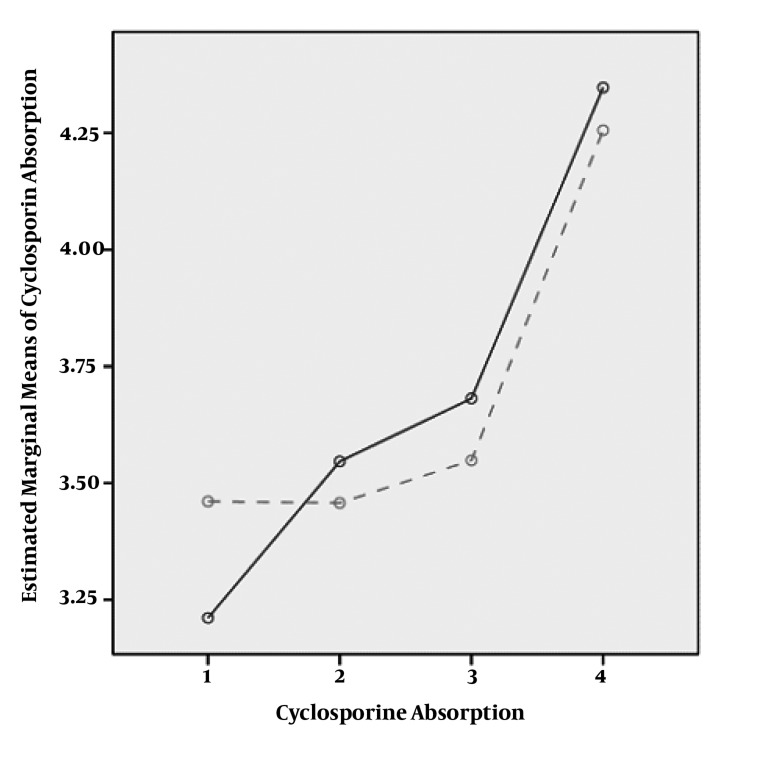
Cyclosporine Absorbtion Change Among Men and Female Throughout a Four Time Patient Follow up.

In our patients, measurement of C0 and C2 and CA levels was repeated after changing dose in four periods post transplantation. All patients were long-term transplant patients with decrease in C0 and C2 and increase in CA. Coefficient of variation for more than four repeated measurements with dose change was 57.1% (C0), 65.4% (C2) and 128% (C2/C0). CA trend was increasing through time (P = 0.00). C0 correlation level was different among males and females (P = 0.00) but C2 and CA were has not different trend in both genders (P = 0.27) in male and (P = 0.83) in female (Figure 1).

### 4.5.Linear Regression Model

Overall CA levels had correlation with lower age of recipient, hypokalemia, higher level of Cr and TG concentrations (Table 3).

**Table 3:** Overall CA Levels and Correlation With other Variable in linear Regression Model

**Time**	***P* value**	**β**	**S.E a**
First three months			
Cr a	0.02	0.177	0.114
Second Year			
Age of Recipient	0.02	-0.005	0.002
TG a	0.001	0.003	0.001
Third year			
Age of Recipient	0.000	-0.003	0.001
K	0.001	-0.091	0.029

a Abbreviations: SE, standard error; Cr, Creatinine, TG, Triglgceride

## 5. Discussion

The present study shows that CsA absorption changes through the post-transplant time and appears to increase over time in long-term period after kidney transplantation. Buchler et al. reported that although CsA dose and levels decreased between months 3 and 12, an increase in the ratio of C2 to C0 was seen within this period of renal transplantation (5). Optimized drug level early after transplantation is still a problem for a significant proportion of kidney transplant recipients (10). Although it shows that absorption of CsA is highly heterogeneous immediately post-transplant period, it increases early after kidney transplantation allowing a reduction in CsA dose to achieve constant exposure (5, 7). Furthermore, we found that CsA levels significantly decrease over first three post-transplant years. Buchler et al. have also shown that C0 and C2 blood levels decreased between months 3 and 12 after kidney transplantation (5). In an international study, CsA doses were decreased over the time while the absorptions of drug were increased (11). Most of studies show that CsA dose reduction is safe in long-term transplant patients (2, 12, 13), and this may be because of improvement in drug absorption. However, a wide alteration in drug absorption is seen among renal transplant recipients due to variable pharmacokinetics of CsA. Effect of concomitant medication and foods are also important (3).

It is clear that greater initial CsA doses to achieve the therapeutic concentrations immediately after transplantation is required to prevent acute rejection (10). In addition, this requirement to higher initial doses of drug may be partly due to the low initial CsA absorption and the low relative bioavailability early after transplantation. Felipe et al reported a significant increasing time-dependent in steady state of CsA exposure occurred within the first month of kidney transplantation (14). It has been reported that a high intra-individual variability of C0 concentrations is associated with a greater incidence of acute and chronic rejection episodes, reduced 5-year graft survival and increased serum creatinine (15). The CsA absorption phase is important for inhibition of T-cell that is maximally achieved when there is an adequate amount of CsA exposure in the early post-dose period (3). The amount of T-cell inhibition relate to the concentration of existing CsA to enter the T cells (3). Variations in CsA absorption have a key role in avoiding overuse of CsA which is prone to nephrotoxicity (3). Although the CsA absorption increases during the early phase after surgery, it may be mainly related to drug accumulation after several dose administrations, and the further CA increases is observed afterward it is most likely correlated to time-dependent changes in relative CA. Interestingly, increase in CA was seen among our patients during three post-transplant years, it means that an increase in drug absorption occurred over the time. This also suggests that the initial higher doses of CsA day would be sufficient to produce therapeutic concentrations for only immediate phase after transplantation. In fact, the relative bioavailability CsA increases over time (16, 17).

The CA of oral CsA administration is usually decreased after transplant surgery, that is parallel to the type and length of anesthesia, hydration, and decreased intestinal motility (18). This increased trend may be attributed to a progressive increase in CA from small intestine and/ or a progressive enhance in CsA metabolism. In a study which revealed that CYP3A4 and PGP activity decreases over time post-transplant (19). The window of CA in the gastrointestinal tract, and differences in content and activity of CYP3A4 and P-glycoprotein may still significantly have control on the absorption of CsA from the micro-emulsion formulation (20-23). Consequently, higher CsA doses early after transplantation are necessary if beneficial concentrations are to be aimed.

The mechanisms involved in this effect are not fully implicated yet (17, 24). In rats, blood CsA absorption get higher after daily administration and are associated with a reduction in hepatic P450 protein expression and microsomal metabolic activity, which suggests that time-dependent P450 suppression by CsA may explain, at least in part, the experiential time-dependent variations in CsA pharmacokinetics (23, 25, 26).

We found no gender difference among CsA absorption; which resembles with the previous finding (27). Roza et al. showed that awareness of the concerns that some patients might absorb CsA differently than others (28), potential differences between gender, ethnicity, and the presence of diabetes were not seen. They demonstrated that male and female kidney transplant recipients responded in the same manner (27). In addition, we found that age may significantly effect CA. Although some studies have shown that age of donor and recipient both are risk factors for acute and chronic rejection episode (29-32), other studies reported that in this group of patients, CsA level tend to be lower (31). However, Sugiyama et al. (32) demonstrated that pharmacodynamic parameters of immunosuppressive drugs did not correlate with age of renal transplant recipients (33). An experimental study on rabbits showed that renal impairment has an important effect on CsA blood levels (34). On the other hand, some other studies showed that CsA dose have linear relation to serum creatinine concentration (27, 33). It is assumed that the decrease in creatinine clearance can result in accumulation of CsA, especially in C2 level, and therefore CA increases during time (35).

Some studies revealed that both CsA nephropathy and hypokalemia may result in renal injuries by chronic vasoconstriction and in combination (36), which is confirmed by our finding. The effect of triglycerides on CA was our another finding of this study, CsA leads to rise in serum LDL and serum free fatty acid level because of insulin resistance and also reduce number and function of hepatic LDL receptors; thus, it seems that CsA and especially higher level of CA may result in accumulation of triglycerides (37-39).

We conclude that it’s better to establish a new marker of CA with regards to absorption profile to avoid any nephrotoxicity because of CsA. Investigation showed that neither C0 nor C2 levels seem to be associated with clinical events, and C2 levels seemed to better reflect inter-individual absorption differences. They may help to identify those patients at risk of over-immunosuppression due to good CA absorption and may enable the CA dose to be lowered in those individuals (3). To our knowledge, an increase observed in the C2 to C0 blood levels ratio during 3 years after renal transplantation, which indicate improvement of CA over time, have not been reported elsewhere. We also conclude that C2/C0 ratio can be useful to select optimal CsA doses in both the early and late post-transplant periods.
